# Changes in Scoliosis Patient and Parental Assessment of Mental Health in the Course of Cheneau Brace Treatment Based on the Strengths and Difficulties Questionnaire

**DOI:** 10.1007/s10882-012-9310-4

**Published:** 2012-11-14

**Authors:** Maciej Glowacki, Ewa Misterska, Katarzyna Adamczyk, Joanna Latuszewska

**Affiliations:** 1 Department of Pediatric Orthopaedics and Traumatology, Poznan University of Medical Sciences, ul. 28 Czerwca 1956 135/147, 61-545 Poznan, Poland; 2 Department of Human Development Psychology and Family Studies, Adam Mickiewicz University, ul. Szamarzewskiego 89, 60-568 Poznan, Poland; 3 Department of Motor System Rehabilitation, Poznan University of Physical Education, ul. Królowej Jadwigi 27/39, 61-871 Poznań, Poland

**Keywords:** Longitudinal analysis, AIS, Brace treatment, Spinal deformity, Mental health, SDQ-25

## Abstract

In the presented study we aimed to investigate the influence of bracing time on perception of mental health by both parents and children with Adolescent Idiopathic Scoliosis (AIS) longitudinally, in relation to socio-demographic and scoliosis-related data. The study design was comprised of three questionnaire assessments, with the second and the third evaluation taking place 6 and 12 months after the beginning of the study, respectively. Thirty-six AIS females treated conservatively and their parents completed the Polish versions of The Strengths and Difficulties Questionnaire-25 (SDQ-25). The results indicated psychiatric disorder was unlikely, but concern all SDQ-25 parent and patient domains and general results. Patient results do not differ significantly in regards to the total score and the particular domains of the SDQ-25. Parents SDQ-25 results differ significantly in regards to the emotional symptoms domain only (*p* = .023, after Bonferroni correction, the difference is insignificant). The study groups differ significantly within the 2nd evaluation in regards to hyperactivity/inattention domain (*p* = .026) and within the last evaluation in regards to emotional symptoms domain (*p* = .009). After Bonferroni correction the differences are insignificant. In general, parents and their children with AIS perceived patients’ mental health in a similar way. Patient and parent assessment of mental health was unchanged after a 12-month brace treatment period. Poor psychological outcome was associated with more severe spinal deformity, brace-wearing duration and age of patient.

## Introduction

Whether present at birth or acquired later in life, a visible disfigurement can have a profound psychological impact upon the individual concerned. The difficulties mentioned above usually include adverse effects on body image, quality of life, and self-esteem (Rumsey and Harcourt 2004). Furthermore, as a lot of research indicates, the experience of disfigurement is multifaceted, involving individual and societal factors, whereas the adjustment process involves the way in which affected individuals interpret their disfigurement, the self, and encounters with others in the long-term perspective (Thompson and Kent 2001).

### Adolescent Idiopathic Scoliosis (AIS)

Adolescent Idiopathic Scoliosis represents a rare condition, classified according to the age of the patient at the time of diagnosis, with a potentially detrimental impact on young patients. Among adolescents its prevalence is 1 to 2 %. AIS is defined as a three-dimensional spinal deformity that affects the coronal, sagittal, and axial planes. The coronal plane deformity is represented on Anterior/Posterior (AP) spine x-rays (radiographs) (Kane 1977; Reamy and Slakey 2001; Trobisch et al. 2010).

To date, the etiology of idiopathic scoliosis is unknown and presumably multifactorial (Miller 1999). Considering sex distribution of idiopathic scoliosis, the fact that male and female infants are equally affected by infantile scoliosis is supported by research, however, girls tend to be more commonly affected with increasing age, so that the sex ratio from age 10 onward is 6:1 (Dobbs and Weinstein 1999; Lonstein 1994; Reamy and Slakey 2001; Roach 1999; Trobisch et al. 2010).

The severity of scoliosis is usually expressed by the Cobb angle, thoracic apical translation and angle of trunk rotation. The Cobb angle is calculated by taking the two vertebral bodies that are most markedly tilted from the horizontal - known as the end vertebrae. A Cobb angle of more than 10° is considered as pathological. The thoracic apical translation is defined as the distance in centimeters between the center of the thoracic apical vertebra and the center central sacral vertical line (CSVL). Another clinical measure of spinal deformity is angle of trunk rotation, determined with a scoliometer (Lonstein 1994; Oestreich et al. 1998; Reamy and Slakey 2001; Trobisch et al. 2010).

The therapeutic goal in children and adolescents is to prevent progression. Scoliosis of less than 20° should be followed up every 4 to 6 months and rechecked with X-rays if progression is suspected. Treatment with a brace is recommended for scoliosis between 20° and 45°. The primary goal of brace treatment is to stop the progression of the spinal curve. Studies conducted on the number of hours per day of brace-wearing usually show that the more hours per day the brace is worn, the better the result. However, it was also noted that the results of 12 h per day of bracing were similar to the results of 23 h per day of bracing. It is usually prescribed between 12 and 23 h a day (Peltonen et al. 1988; Takemitsu et al. 2004). Surgical correction and stabilization of the affected segment of the spine is considered for the treatment of adolescent scoliosis exceeding 45° (Reamy and Slakey 2001).

### Psychosocial and Family Aspects of Scoliosis-Related Deformity and Treatment Interventions

Considering the psychosocial effects of scoliosis-related deformity and treatment interventions, some retrospective studies have provided little support for evidence of psychosocial impairment due to scoliosis, while others have found that AIS is related to serious disturbances in mental health or body image disorders in particular (Apter et al. 1978; Brosnan 1991; Tones et al. 2006). General conclusions were that bracing may cause some psychological stress to the patient, at least at the initiation of treatment and possibly in the long-term period (Donnelly et al. 2004). MacLean et al. (1989) discovered the majority of patients experience a significant period of stress and decrease of self-esteem at the initiation of conservative brace treatment (88 %). They found no evidence of overt psychopathology but did describe some of the issues patients face with brace-wearing such as soreness, discomfort with activity, difficulty in finding clothes and difficulty in social interaction. What is more, in a study by Donelly et al. (2004) conservatively treated patients reported feelings of being singled out because of the illness and outward appearance.

Furthermore, the conflicts patients and parents discussed regarding brace-wearing were of special significance to participants as they reflected the peer pressure experienced to conform and coexisting pressure from parents to wear a brace, therefore, to be different (Donnelly et al. 2004). The ability of parents to understand the perspective of the pediatric orthopedic patient has been extensively studied and results indicate a strong level of agreement between child and parent responses in general. However, significant discrepancies regarding parental and patient evaluations of the physical functioning, general health and mental health of adolescents have been confirmed (Matsumoto et al. 2011). Similarly, a study by Rinella et al. (2004), and Smith et al. (2006) supported the notion that trunk deformity in AIS can be perceived differently by children and parents. Since the development of body image disturbances may be influenced by negative family attitudes toward adolescents or hearing critical comments about one’s trunk deformity (McCabe and Ricciardelli 2003; Viviani 2006), it is necessary, in our opinion, to consider the discrepancies regarding patient and parental perceptions of AIS and its psychosocial effects. What is more, we believe a detailed longitudinal exploration of the dynamics of patient psychological impairment due to body disfigurement and brace treatment, from the perspective of both AIS females’ and their parents, may shed new light on factors determining patients’ functioning and, therefore, constitute useful implications for spine clinicians.

Taking the above mentioned assumptions and inconsistencies of the study results into consideration, a longitudinal, detailed analysis of discrepancies between parents’ and female adolescents’ with scoliosis, in terms of The Strengths and Difficulties Questionnaire-25 (SDQ-25) (Goodman 1997, 2001) areas of clinical interest such as hyperactivity/inattention, emotional symptoms, conduct problems, peer relation problems and pro-social behavior, is necessary. In particular, our study design is aimed to investigate the influence of bracing time on the perception of health held by adolescents with a diagnosed visible trunk deformity and their parents, in relation to socio-demographic and scoliosis-related data.

## Material and Methods

### Study Design and Research Procedures

The study design was prospective and comprised of three clinical and questionnaire assessments. The radiological evaluation was performed during the first and the third assessment. The mental health perception of both patients and parents was evaluated based on the subscales of the SDQ-25. Polish versions of the self-report and parent report SDQ-25 were used at the first visit, and then 6 months and again 12 months after the beginning of the study (Mazur et al. 2007). Both SDQ-25 versions were completed during a routine patient visit. The investigator was available during this time if study participants required any explanation or clarification. All patients received detailed information on the aim of the study and were assured of anonymity, following which they gave their informed consent. The study was approved by the Bioethics Committee.

### Selection Criteria of Study Sample

The study group was comprised of 36 consecutively selected female patients with AIS treated conservatively with a Cheneau brace and their parents. The inclusion criteria of female patients were as follows: a minimum duration of Cheneau brace application of at least 12 h per day, a Cobb angle of 20–40°, 10 to 17 years of age. We excluded patient in whom other diseases leading to trunk deformity or serious medical conditions were diagnosed.

### Characteristics of Scoliosis Patients

Table 1 presents detailed clinical and socio-demographic characteristics of scoliosis patients. Twenty-one patients (58.00 %) had thoracic scoliosis, 12 patients (33.00 %) had thoraco-lumbar scoliosis, and the remaining 3 patients (9.00 %) had lumbar scoliosis. Th8 was the apical vertebra in ten patients; Th9 in four patients; Th10 in two patients; Th11 in five patients; Th12 in five patients. L1 was the apical vertebra in seven patients; L2 in one, and L3 in two patients.

**Table 1:** Characteristics of Study Participants

Parameters	1st Evaluation		2nd Evaluation		3rd Evaluation	
	Mean (SD)	Range (Min-Max)	Mean (SD)	Range (Min-Max)	Mean (SD)	Range (Min-Max)
Body Mass Index	17.40 (1.07)	12.90–21.00	17.80 (1.90)	13.70–22.20	18.10 (1.90)	14.00–22.70
Age [Years]	13.40 (1.70)	10.00–16.00	14.00 (1.90)	1.10–20.17	14.40 (1.70)	11.00–18.00
Cobb Angle	27.10 (5.10)	20.00–38.00	---	---	24.90 (9.10)	5.00–48.00
Angle of Trunk Rotation^a^	8.30 (3.10)	3.00–15.00	7.80 (3.50)	1.00–15.00	6.90 (3.50)	2.00–15.00
Apical Translation [cm]^b^	2.10 (0.90)	0.40–4.00	---	---	2.10 (1.00)	0.20–4.20
Brace [Hours/Day]	15.90 (2.90)	8.00–22.00	15.60 (2.60)	9.00–22.00	15.20 (2.20)	9.00–18.00
Brace [In Months]	17.90(17.60)	1.00–69.00	24.50 (17.50)	5.00–76.00	30.10 (17.60)	12.00–81.00

^a^Angle of trunk rotation as measured with Perdriolli’s inclinometer

^b^The degree of the apical translation of the central sacral vertical line (CSVL) according to the Harms Study Group

During the first, second and the last evaluation patients were assessed at a mean of 17.90 months, *SD =* 17.60, 24.50 months, *SD* = 17.50, and 30.10 months, *SD =* 17.60, respectively, after beginning Cheneau brace treatment. They wore the brace for 15.90 h, *SD* = 2.90, 15.60 h, *SD* = 2.6 and 15.20 h*, SD* = 2.20 a day on average, respectively. Patients’ age was 13.40 year, *SD* = 1.70 in the first assessment, 14.00 year, *SD* = 1.90 in the second, and 14.40 year, *SD* = 1.70 in the last evaluation. The mean Cobb angle was 27.10°, *SD* = 5.10 in the first and 24.90°, *SD* = 9.10 in the final examination.

### Measurement Instruments

#### SDQ-25 Background Information

The SDQ-25, designed by Goodman in 1997, is a structured questionnaire which is commonly used for behavioral and emotional problems as well as assessing the relationships of children and adolescents (Goodman 1997). The questionnaire includes 25 questions covering five areas of clinical interest: emotional problems, hyperactivity, peer problems, conduct problems or pro-social behavior. There are five items per area. The SDQ-25 appeals to researchers and clinicians for several reasons: firstly, because of its brevity, secondly because it covers key aspects of common childhood and adolescence psychopathology, and thirdly because it includes strengths as well as difficulties, which makes it more acceptable for parents, especially in the general population (Goodman 1997; Mazur et al. 2007). There are three different versions of the questionnaire: the parent version and teacher version covering all ages and a self-reported version which is used only among adolescents. Each item of SDQ-25 can be answered as “Not True”, “Somewhat True” and “Certainly True”, each answer is scored from 0 to 2 points where ‘not true’ receives either 0 or 2 depending on the template (Goodman 1997, 2001).

#### Psychometric Properties of SDQ-25

The SDQ has been translated into more than 60 languages and its psychometric properties have been evaluated in many countries. According to Goodman’s findings (1997), the Cronbach’s alpha coefficient for the different scores and informants are generally satisfactory (mean .73). Another study by Goodman (2001) demonstrated a mean retest stability of .62 for the parent-rated SDQ despite a very long interval of 4 to 6 months. Teacher ratings were most stable (mean correlation .73) and youth ratings least stable (mean correlation .51). Considering the factor structure of the original version of the SDQ-25, a five-factor structure was confirmed by using an exploratory factor analysis for the parent, teacher, as well as self versions (Goodman 2001). Considering criterion-related validity, high SDQ-25 values were found to be connected with a considerably higher risk of a relevant DSM-IV diagnosis. Children and adolescents whose parent or teacher SDQ total difficulties scores placed them in the top 10 % of the population (“abnormal range”) were 15 times more likely to have a DSM-IV diagnosis; for self report, the corresponding risk was 6 times higher (Goodman 2001)

Considering the psychometric properties of the Polish version of SDQ-25, the factor analysis suggested a four-component structure of SDQ (Emotional, Conduct, Hyperactivity, Peers), which explains 41.60 % of total variance. The Cronbach’s alpha value equaled .76. for the SDQ-25 total score, .67 for emotional symptoms, .38 for conduct problems, .55 for hyperactivity and .46 for interpersonal relationships (Mazur et al. 2007).

#### Scoring and Interpretation of the SDQ-25 Results

The score for each of the subscales is obtained adding the points of the five items from each subscale, thus generating a score which ranges from 0 to 10. The scores from the subscales of hyperactivity, emotional problems, conduct and relationship are added together generating a total score of difficulties ranging from 0 to 40. The points from the pro-social scale are not incorporated in the total score of difficulties, as the absence of pro-social behavior is conceptually different from the presence of psychological difficulties. The published cut-off points which are available at www.sdqinfo.com were used to define “normal” or “psychiatric disorder: unlikely”, “borderline” or “psychiatric disorder: possible” and “abnormal” or “psychiatric disorder: probable” scores (Goodman 1997; Mazur et al. 2007).

#### Statistics

In respect to statistical quantitative features, the means, 95 % confidence intervals, range and standard deviations were determined. In respect to qualitative features, the number of units that belong to the described categories of a given feature and their relative percentage values were given. The Friedman two-way ANOVA test was used for multiple follow-up SDQ-25 comparisons. Wilcoxon signed ranks tests were used to analyze discrepancies between the patients’ and their parents’ SDQ-25 results. To protect against Type I errors, a Bonferroni adjustment for multiple comparisons was made. Spearman’s rank order correlation coefficients were applied to evaluate correlations between quantitative variables. The logistic regression analysis was used to evaluate the influence of the socio-demographic, brace-related and the radiological data on the probability of achieving a “normal” result in the SDQ-25 questionnaire. Based on the adopted cut-off points (Goodman 1997, 2001; Mazur et al. 2007), a total score between 0 to 13 points in the SDQ-25 parent version was considered a “good result”, whereas scoring between 14 to 40 points was a “poor result”. In the SDQ-25 patient version, a total score below 15 points was considered a “good result” while scoring between 16 to 40 points was a “poor result”. The accepted border level of statistical significance was *p* = .05 and therefore any test results where the p value exceeded this level were treated as insignificant. Statistical calculations were performed by means of Statistica software.

## Results

### Descriptive Statistics of the Strengths and Difficulties Questionnaire-25 for Patients

Tables 2 and 3 present a detailed analysis of the distribution of scores for both the SDQ-25 parent and patient versions: the minimal and maximal values, mean scores and 95 % confidence intervals. The results are presented for individual domains as well as for the total scores. According to the accepted cut-off points (Goodman 1997, 2001; Mazur et al. 2007), all SDQ-25 domain and general results obtained an overall result indicating “psychiatric disorder: unlikely” in both the patient and parent versions. Our study achieved a “normal” result for the 1st, 2nd and 3rd assessments (for details see Table 2 and Table 3).

**Table 2:** Means, Maximum and Minimum Values, and Standard Deviations of SDQ-25 Scores: Patients

SDQ-25 Subscales	1st Evaluation			2nd Evaluation			3rd Evaluation		
	Mean (SD)	95 % Confidence Interval (From-To)	Range (Min-Max)	Mean (SD)	95 % Confidence Interval (From-To)	Range (Min-Max)	Mean (SD)	95 % Confidence Interval (From-To)	Range (Min-Max)
Emotional Symptoms	2.06 (1.55)	1.53–2.58	0.00–5.00	1.89 (1.70)	1.31–2.47	0.00–5.00	1.86 (1.66)	1.30–2.42	0.00–6.00
Conduct Problems	1.47 (1.06)	1.12–1.83)	0.00–5.00	1.47 (1.18)	1.07–1.87	0.00–5.00	1.44 (1.21)	1.04–1.85	0.00–5.00
Hyperactivity/Inattention	2.56 (1.95)	1.90 (3.21)	0.00–7.00	2.36 (1.50)	1.86–2.87	0.00–7.00	2.11 (1.75)	1.52–2.70	0.00–6.00
Peer Relation Problems	1.25 (1.08)	0.88 (1.62)	0.00–5.00	0.94 (1.17)	0.55–1.34	0.00–6.00	1.03 (1.21)	0.62–1.44	0.00–4.00
Pro-social Behavior	2.19 (1.67)	1.63 (2.76)	0.00–6.00	2.03 (1.40)	1.55–2.50	0.00–5.00	2.11 (1.56)	1.58–2.64	0.00–6.00
Total Score	7.33 (3.53)	6.14 (8.53)	1.00–15.00	6.67 (4.30)	5.21–8.12	0.00–23.00	6.44 (4.31)	4.99–7.90	0.00–18.00

*SDQ-25* The Strengths and Difficulties Questionnaire-25

**Table 3:** Means, Maximum and Minimum Values, and Standard Deviations of SDQ-25 Scores: Parents

SDQ-25 Subscales	1st Evaluation			2nd Evaluation			3rd Evaluation		
	Mean (SD)	95 % Confidence Interval (From-To)	Range (Min-Max)	Mean (SD)	95 % Confidence Interval (From-To)	Range (Min-Max)	Mean (SD)	95 % Confidence Interval (From-To)	Range (Min-Max)
Emotional Symptoms	2.02 (1.74)	1.43–2.62	2.00–0.00	1.58 (1.46)	1.09–2.078	0.00–5.00	1.17 (1.83)	0.55–1.79	0.00–6.00
Conduct Problems	1.52 (1.56)	1.00–2.05	1.00–0.00	1.19 (1.06)	0.83–1.56	0.00–4.00	1.28 (1.19)	0.88 (1.68)	0.00–5.00
Hyperactivity/Inattention	2.06 (2.06)	1.36–2.75	1.50–0.00	1.67 (1.35)	1.21–2.12	0.00–5.00	1.58 (1.61)	1.04 (2.13)	0.00–5.00
Peer Relation Problems	1.64 (1.76)	1.04–2.23	1.00–0.00	1.08 (1.11)	0.71–1.46	0.00–4.00	1.03 (1.00)	0.69 (1.37)	0.00–3.00
Pro-social Behavior	1.67 (1.84)	1.05–2.29	1.00–0.00	1.64 (1.69)	1.07–2.21	0.00–6.00	1.69 (1.65)	1.14 (2.25)	0.00–5.00
Total Score	7.25 (6.01)	5.22–9.29	5.50–0.00	5.53 (3.78)	4.25–6.81	0.00–14.00	5.06 (3.58)	3.85 (6.27)	0.00–13.00

*SDQ-25* The Strengths and Difficulties Questionnaire-25

In the patient questionnaires from the 1st assessment, we obtained the following amount of children with low scores, falling within the normal range: 100.00 % (36 patients) in the subscale of emotional symptoms, 97.20 % (35 patients) for conduct problems, 91.70 % (33 patients) for hyperactivity, 97.20 % (35 patients) for interpersonal relationships, 88.90 % (32 patients) for pro-social behavior and 100.00 % (36 patients) for the total score. The second assessment patient questionnaires provided 100.00 % (36 patients) in the subscale of emotional symptoms, 94.40 % (34 patients) for conduct problems, 94.40 % (34 patients) for hyperactivity, 97.20 % (35 patients) for interpersonal relationships, 91.70 % (33 patients) for pro-social behavior and 97.20 % (35 patients) for the general result. The last assessment of patient questionnaires delivered 97.20 % (35 patients) of children falling within the normal range in the subscale of emotional symptoms, 94.4 % (34 patients) for conduct problems, 94.40 % (34 patients) for hyperactivity, 94.40 % (34 patients) for peer relations, 88.90 % (32 patients) for pro-social behavior and 94.40 % (34 patients) for the total score (for a detailed distribution of “borderline” and “abnormal” SDQ-25 patient scores see Table 4).

**Table 4:** Distribution of “Normal”, “Borderline” and “Abnormal” SDQ-25 Scores: Patients and Parents

SDQ-25 Subscales^a^	1st Evaluation			2nd Evaluation			3rd Evaluation		
	A (%)	B (%)	C (%)	A (%)	B (%)	C (%)	A (%)	B (%)	C (%)
Emotional Symptoms (Patient/Parent)	100.00/77.80	0.00/13.90	0.00/8.30	100.00/86.10	0.00/11.10	0.00/2.80	97.20/83.30	2.80/5.60	0.00/11.10
Conduct Problems (Patient/Parent)	97.20/86.10	2.80/2.80	0.00/11.10	94.40/88.90	0.00/8.30	5.60/2.80	94.40/88.90	0.00/2.80	5.60/8.30
Hyperactivity/Inattention (Patient/Parent)	91.70/91.70	2.80/5.60	5.50/2.70	94.40/100.00	2.80/0.00	2.80/0.00	94.40/100.00	5.60/0.00	0.00/0.00
Peer Relation Problems (Patient/Parent)	97.20/75.00	2.80/5.60	0.00/19.40	97.20/86.10	2.8.00/11.10	0.00/2.80	94.40/88.90	5.60/11.10	0.00/0.00
Pro-social Behavior (Patient/Parent)	88.90/91.70	8.30/2.80	2.80/5.50	91.70/91.70	8.30/5.60	0.00/2.70	88.90/94.40	8.30/5.60	2.80/0.00
Total Score (Patient/Parent)	100.00/80.60	0.00/11.10	0.00/8.30	97.20/97.20	0.00/2.80	2.80/0.00	94.40/100.00	5.60/0.00	0.00/0.00

*A* psychiatric disorder: unlikely; *B* psychiatric disorder: possible; *C* psychiatric disorder: probable; ^a^patients/parents; *SDQ-25* The Strengths and Difficulties Questionnaire-25

### Descriptive Statistics of the Strengths and Difficulties Questionnaire-25 for Parents

The questionnaires rating AIS patient mental health, answered by parents in the 1st assessment, provided the following percentages of children with low scores (falling within normal): 77.80 % (28 patients) in the emotional symptoms subscale, 86.10 % (31 patients) for conduct problems, 91.70 % (33 patients) for hyperactivity, 75.00 % (27 patients) for interpersonal relationships, 91.70 % (33 patients) for pro-social behavior and 80.60 % (29 patients) for the total score. Questionnaires answered by parents in the 2nd assessment showed “normal” results for 86.10 % of children (31 patients) in the subscale of emotional symptoms, 88.90 % (32 patients) for conduct problems, 100.00 % (36 patients) for hyperactivity, 86.10 % (31 patients) for interpersonal relationships, 91.70 % (33 patients) for pro-social behavior and 97.20 % (35 patients) for the general result. In the last assessment 83.30 % of children (30 patients) fell within the normal range in the emotional symptoms subscale, 88.90 % (32 patients) for conduct problems, 100.00 % (36 patients) for hyperactivity, 88.90 % (32 patients) for peer relations, 94.40 % (34 patients) for pro-social behavior and 100 % (36 patients) for the total score (for a detailed distribution of “borderline” and “abnormal” SDQ-25 parent scores see Table 4).

### Multiple Comparisons Between the 1st, 2nd and 3rd SDQ-25 Results

Table 5 presents the results of multiple comparisons between the 1st, 2nd and 3rd evaluations by means of SDQ-25 patient and parent forms, both for the general results and for individual domains. Patient results do not differ significantly in regards to the total score and the particular domains of the SDQ-25. Parents SDQ-25 results differ significantly in regards to the emotional symptoms domain only (*p* = .023), (for details see Table 6). However, following Bonferroni adjustment for 6 comparisons and the adoption of *p* = .008 as the border level of statistical significance, the indicated differences became statistically insignificant.

**Table 5:** Results of Multiple Comparisons between the 1st, 2nd and 3rd SDQ-25 Patients and Parents Results

SDQ-25 Subscales	SDQ-25-Patient Form	SDQ-25-Parent Form
	*p*	*p*
Emotional Symptoms	.674	.023
Conduct Problems	.776	.228
Hyperactivity/Inattention	.521	.837
Peer Relation Problems	.110	.250
Pro-social Behavior	.621	.968
Total Score	.245	.071

*SDQ-25* The Strengths and Difficulties Questionnaire-25

**Table 6:** Results of Cross-Group Comparisons between The SDQ-25 Patients and Parents Results

SDQ-25 Subscales	1st Evaluation	2nd Evaluation	3rd Evaluation
	*p*	*p*	*p*
Emotional Symptoms	.814	.515	.009**
Conduct Problems	.537	.358	.481
Hyperactivity/Inattention	.201	.026*	.155
Peer Relation Problems	.709	.530	.726
Pro-social Behavior	.112	.137	.221
Total Score	.309	.235	.152

*SDQ-25* The Strengths and Difficulties Questionnaire-25

*p < .05; **p < .01

### Cross-Group Comparisons Between the SDQ-25 Patient and Parent Results

Table 6 presents results of cross-group comparisons of the SDQ-25 between patient and parent subgroups, both for the general results and for individual domains. The study groups differ significantly in the 2nd evaluation in regards to the hyperactivity/inattention domain (*p* = .026) and parents scored lower. In the last evaluation there was a significant difference in the emotional symptoms subscale (*p* = .009), with parents obtaining lower scores. However, after Bonferroni adjustment, the indicated differences became statistically insignificant.

### Associations Between Patient Characteristics and AIS Patient and Parental Rates of Mental Health

Having analyzed the correlation between patient characteristics and patients/parents results in the 1st evaluation, we indicated a weak association between patient perception of peer relation problems and angle of trunk rotation (r*s* = .30, *p* = .049) and between parent assessment of patient peer relations and age of patients (r*s* = .39, *p* = .018).

In the 2nd assessment we revealed that the only significant, although weak correlations concerned patient assessment of hyperactivity/inattention and brace wearing in months (r*s* = .34, *p* = .040) and between parent perception of pro-social behavior and BMI (r*s* = .37, *p* = .027). A moderate association between parental assessment of pro-social behavior and age was also visible (r*s* = .50, *p* = .002).

In the last evaluation there is a weak correlation between patient (r*s* = .38, *p* = .021) and parent (r*s* = .37, *p* = .025) assessment of peer relation problems and brace wearing per day as well as parent assessment of patient emotional symptoms and apical translation (r*s* = .35, *p* = .036) and brace-wearing in months (r*s* = .46, *p* = .005).

### Associations Between Patient-Parent SDQ-25 Total Score Differences and Patient Characteristics

We have analyzed the relation between patient-parent SDQ-25 total score differences and patient characteristics. To analyze if a younger age led to disparities between parent and patient perceptions of mental health, Spearman’s correlations of patients’ age with patient–parent total score differences were performed. Correlations of Cheneau brace application (daily and monthly) and clinical and radiographic scoliosis parameters with patient–parent total score differences were applied to verify if patient–parent perspectives correlate better as bracing time or severity of spinal deformity increases. Our analysis revealed that in the first assessment the Cobb angle was related to the differences in perception of patient emotional symptoms (r*s* = .35, *p* = .034); during the second evaluation there is a relation between age of patients and the difference in patient-parent assessment of pro-social behavior (r*s* = −.50, *p* = .002). Moreover, we indicated the angle of trunk rotation was related to the difference in patient-parent general perception of mental health in the last evaluation (r*s* = .36, *p* = .003).

### Regression Analysis

The logistic regression analysis aimed to evaluate the influence of the brace-wearing related, socio-demographic and the radiological data on the probability of achieving a “good result” in the SDQ-25 patient and parent forms, could not be performed due to the distribution of the results in which almost all of the participating patients and parents obtained a “good result” score in the general result of SDQ-25, in all the evaluations carried out.

## Discussion

The objective of the present study was to screen for possible psychopathological impairment among females with AIS in the course of a 12-months orthosis treatment period by means of the SDQ-25. This assessment tool permitted the investigation of the relationships between emotional problems, hyperactivity, peer problems, conduct problems or pro-social behavior in terms of brace- and scoliosis-related data. To date, no other longitudinal studies have examined these factors and their interrelationships in the course of conservative treatment for AIS. According to our knowledge, no previous study has addressed the association between AIS patients’ self-assessments of mental health outcome and parents’ assessments of their child’s mental health as verified by SDQ-25 questionnaire data.

It must be emphasized that *The Strengths and Difficulties Questionnaire-25* clinical usefulness in order to screen probable psychiatric disorders is well supported (Goodman 1997, 2001; Mazur et al. 2007). Moreover, the questionnaire also indicates the risk factors which influence the psychosocial well-being of children. In our opinion the above mentioned assumptions are of special importance, since patients with a visible disfigurement often experience significant psychosocial problems, in addition to physical and functional difficulties. Additionally, as research indicates, disfigurement appears to have a consistent negative impact on the development of an individual’s body image, and this may result in increases in anxiety, stress, suicidal thoughts, decreased self-esteem and social confidence. Furthermore, people with a disfigurement have an exaggerated tendency to attribute the negative behavior of others to their disfigurement which in turn often leads to a feeling of helplessness, shame, humiliation, embarrassment, hopelessness, and ultimately depression (Roberts et al. 2006). As previously reported, social isolation, depression and reduced leisure activities may be experienced by patients with AIS, especially those subjected to conservative treatment, and the prevalence of psychological disorders in this group of patients may be as high as 19.00 %. Therefore, scoliosis is currently recognized as an important risk factor for psychological discomfort, especially in brace-treated patients (Aulisa et al. 2010). It was concluded that bracing may have a negative psychological impact, especially at the beginning of treatment, evidenced by reduced self-esteem (MacLean et al. 1989). Meanwhile, our study results are contrary to the above mentioned findings, since the majority of AIS patients, from the perspective of both children and parents, are within the normal range when compared to the established norms, indicating good psychosocial adaptation to scoliosis deformity and brace-treatment regimen. Interestingly, after accounting for the significance of differences in the SDQ-25 results between three assumed evaluations, the presented results support previous findings regarding improvement of patient functioning and emotional reactions in particular after the initial period of tension and then in the course of orthosis treatment, as evaluated from the parent perspective. However, it must be emphasized, after Bonferroni correction, the indicated differences became insignificant. Additionally, correlational analysis supported associations between monthly and daily duration of brace-wearing and psychological functioning of AIS patients, especially when considering worsened hyperactivity/inattention, peer relation and emotional problems, as bracing time increases.

Cumulative research evidence suggests that a child with a disfigurement is at increased risk of disturbed attachments to primary caregivers, being the subject of staring, intrusive questioning, ridicule, hostility in public, avoidance, decreased popularity and teasing from peers, impaired social adjustment, social anxiety and behavior problems (Hearst 2007). Patients and parents found the discussion of conflicts regarding brace-wearing were significant since peer pressure to conform exists and patients face parental pressure to wear a brace which in fact makes them stand out (Donnelly et al. 2004). Moreover, as previously stated, spinal deformity in AIS can be perceived differently by children and parents (Rinella et al. 2004; Smith et al. 2006). However, most of the analyzed research accounting for discrepancies between parent and patient perceptions of health and disease focused on surgical treatment outcomes. In this study an interesting association has been confirmed regarding better concordance of parent and patient perspectives as spinal deformity decreases, indicating the significance of scoliosis progression or stabilization for the possible discrepancies between the evaluation of patient situation from both child and parent perspectives.

Considering the possible differences between patient and parent assessments of patient functioning, Matsumoto et al. (2011) indicated children between the ages of 5 and 18 with a wide range of musculoskeletal problems reported a higher level of Physical Functioning and lower levels of both General Health and Mental Health than their parents on the *Child Health Questionnaire*. Additionally, parents reported significantly higher expectations for treatment than their children did (Matsumoto et al. 2011). Zhang et al. (2011) emphasized, in terms of mental health, that conservative treatment is not ideal for patients with mild to moderate scoliosis, and in particular, it is not conducive to mental health in patients with a Cobb angle between 40° and 50°. Therefore, the mental effect that could occur in patients choosing this scoliosis therapy should be considered. Rinella et al. (2004) in a prospective, cross-sectional analysis aimed to determine the correlation of parent and patient perspectives of the patients’ preoperative and postoperative experience using the *Scoliosis Research Society-24* (*SRS-24*) questionnaire emphasizing parent-patient disparities. Based on *SRS-24* data, parents typically scored higher than their children in the operative treatment of idiopathic scoliosis in total score, self-image, and overall satisfaction. Some parent-patient scores correlated better with increasing age of the patient, and later in the postoperative period (Rinella et al. 2004). Meanwhile, in the presented results, we highlighted patients overestimated hyperactivity/inattention and emotional problems when compared to parent opinions, significant differences emerged later in the observed treatment period. However, after Bonferroni correction, the differences became insignificant. Similarly, as in a study of Rinella et al. (2004), the patient-parent perspective, of perception of pro-social behavior in particular, became closer as patient age increased. The discussed findings are consistent with the observations of developmental psychologists that late adolescents increasingly identify with their parents’ values and perspectives in comparison to early adolescents (Boyd and Bee 2004).

Sanders et al. (2003) indicated parents perceive more deformity of the ribs and shoulders than did the patients, but other aspects of the deformity are identified equally, whereas Smith et al. (2006) determined the agreement between patient and parent perceptions of the patient’s postoperative appearance and indicated that patients and parents do not strongly agree on the cosmetic outcome of AIS surgery. Therefore, given that it is the adolescents themselves who undergo surgery, patient assessments of their deformity, rather than radiographic measures or parental assessments, should play a major role in the evaluation of surgical success. Bridwell’s et al. (2000) study of parents and patient concerns and preferences regarding surgery for idiopathic scoliosis indicated parental concerns were higher, and their expectations were greater than those of the patients. Meanwhile, our research regarding parent and patient perceptions of mental health in the course of scoliosis Cheneau brace treatment indicated the score means in most scales were comparable, suggesting strong agreement between both analyzed groups. However, after 6 and 12 months of the observed treatment period, patient self-evaluation of hyperactivity/inattention and emotional symptoms were more severe than did their parents, indicating the overestimation of symptoms from the AIS patient perspective. This discrepancy was, as mentioned above, significant before the Bonferroni correction.

Taking the relationships between the SDQ-25 results and the clinical and radiological assessments of spinal deformity into consideration, it must be emphasized there is little empirical support to date for the relationship between size or severity of disfigurement and psychological distress (Rumsey et al. 2004). As concluded by Smith et al. (2008) and Buchanan et al. (2003) there is a poor association between radiological and clinical assessment performed by doctors, and evaluation of appearance and satisfaction with the treatment by scoliosis patients and their parents. However, our study supports the relationships between patient and parent evaluations of psychological well-being, particularly in terms of emotional symptoms as well as peer relations, and severity of spinal deformity. The presented results are contrary to previous findings suggesting that size and severity of disfigurement are not directly related to the extent of psychological impairment of patient (Buchanan et al. 2003; D'Andrea et al. 2000; Koch et al. 2001; Rumsey et al. 2004; Smith et al. 2008; White et al. 1999).

### Study limitations and future research implications

Some limitations of the present study should be noted. The majority of questionnaire forms were completed by mothers rather than fathers. Furthermore, the current study specifically investigated female patients only, this may have had an impact on the distribution of scores and limit the generalizability of the findings. Therefore, more a comprehensive evaluation of brace treatment should include longitudinally analyzed assessment of mental health from the perspective of male AIS patients and both parents. Furthermore, the current study did not address parent-related socio-demographic factors in determining their assessment of AIS patients mental health. Therefore, future studies would benefit from including the aforementioned variables in data analyses.

Taking into account research highlighting the multivariate nature of adjustment to disfigurement and the role of appearance-specific cognitions in influencing levels of distress (Rumsey and Harcourt 2004), we support the need to further investigate the interrelationships between patients’ perceptions of mental health, spinal appearance and stress level, bearing in mind that many components of adjustment are amenable to psychological intervention. Finally, additional studies could examine the predictive validity of the *The Strengths and Difficulties Questionnaire-25* for future psychological impairment in adult scoliosis patients treated conservatively in adolescence. Moreover, it would serve to identify protective and/or scoliosis-related risk factors for psychological impairment.

## Conclusions

1. In general, parents and their children with AIS perceive patients’ mental health in a similar way, indicating good psychosocial adaptation to disease and applied treatment method.
2. Patient and parent assessment of mental health was unchanged after a 12-month brace treatment period.
3. Poor psychological outcome was associated with more severe spinal deformity, brace-wearing duration and age of patient.
4. Discrepancies between parents’ and AIS females’ assessments of mental health increase with the amount of spinal deformity.
